# Interspecific variation of prevalence by *Scaphanocephalus* (Platyhelminthes: Trematoda: Heterophyidae) metacercariae in parrotfishes (Labridae: Scarini) from an Okinawan coral reef

**DOI:** 10.1016/j.ijppaw.2020.05.007

**Published:** 2020-05-22

**Authors:** Tamaki Shimose, Hirotaka Katahira, Minoru Kanaiwa

**Affiliations:** a Research Center for Subtropical Fisheries, Seikai National Fisheries Research Institute, Japan Fisheries Research and Education Agency, 148, Fukai-Ohta, Ishigaki, Okinawa, 907-0451, Japan; b Department of Environmental Science, School of Life and Environmental Science, Azabu University, 1-17-71 Fuchinobe, Chuo-ku, Sagamihara, Kanagawa, 252-5201, Japan; c Mie University, Faculty of Bioresources, 1577 Kurimamachiya-cho, Tsu City, Mie, 514-8507, Japan

**Keywords:** *Calotomus*, *Cetoscarus*, *Chlorurus*, Metacercariae, Mucous cocoon envelope

## Abstract

Metacercarial cysts of the parasite *Scaphanocephalus* (Platyhelminthes: Trematoda: Heterophyidae) are frequently found on the pectoral fins and skin of parrotfishes (Labridae: Scarini) inhabiting Okinawan coral reefs in southern Japan. The prevalence of metacercarial cysts in 30 parrotfish species was investigated and compared through a market survey. Although parasite prevalence differed between parrotfishes, all species examined are considered to be suitable hosts. Prevalence was high in *Scarus chameleon* (38.5%, n = 13), *S. rubroviolaceus* (33.4%, 2797), *S. ghobban* (26.6%, 6441), and several other species that share, in part, common feeding habits. Conversely, prevalence was low in *S. prasiognathos* (0.4%, 1842), *Bolbometopon muricatum* (0.4%, 270), and *Hipposcarus longiceps* (0.1%, 8512) which have different feeding habits. Despite a few exceptions, feeding ecology and other indirect behaviors are considered to affect the prevalence of metacercarial cysts in parrotfishes. Taxonomic affiliation and nocturnal mucous cocoon usage are not considered to affect parasite prevalence.

## Highlights

• Metacercarial cysts prevalence in parrotfishes was investigated.
• All the species seem to be suitable hosts, but prevalence differed among species.
• Feeding ecology is thought to affect the prevalence in parrotfishes.
• Nocturnal mucous cocoon use does not appear to influence parasite prevalence.

## Graphical abstract

**Figure:**
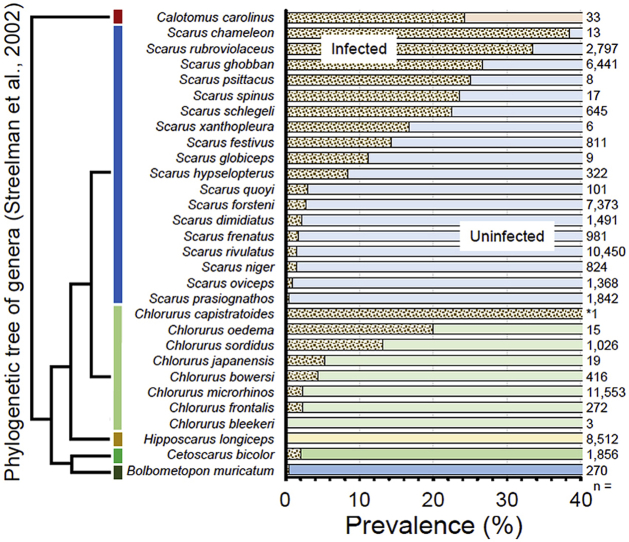

## 1 Introduction

**Fig. 1:**
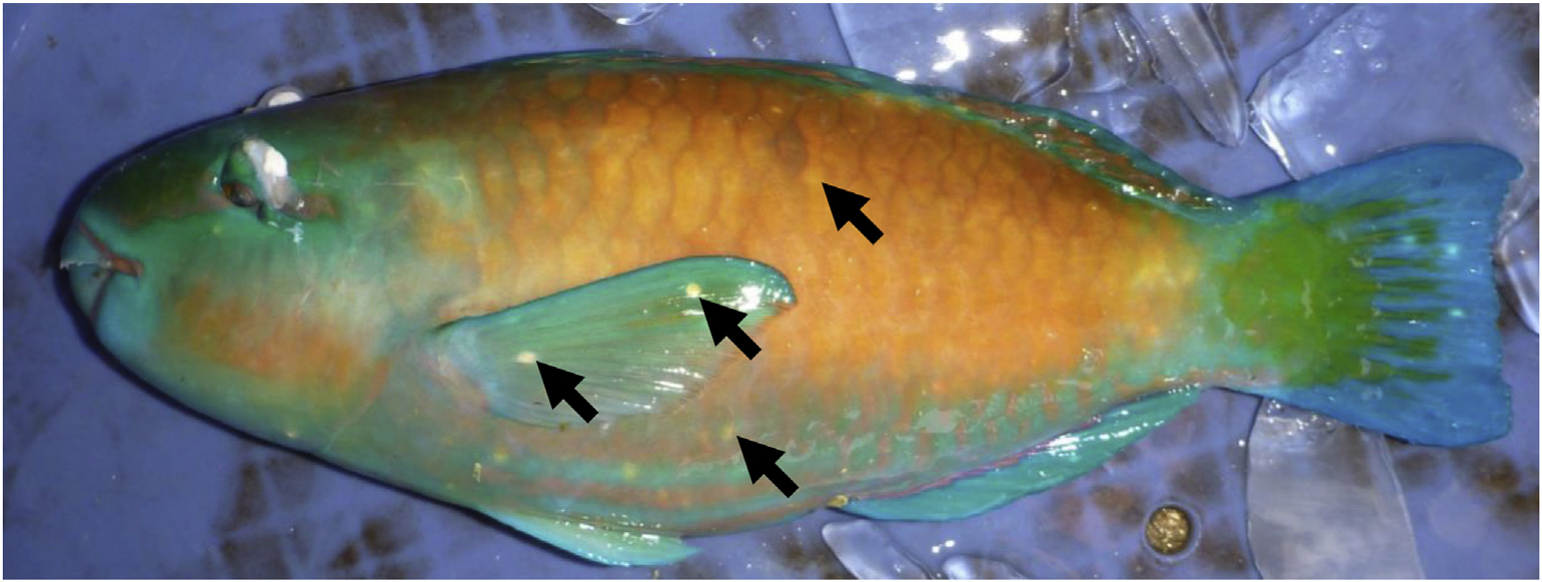
Cyst of *Scaphanocephalus* parasite (arrows) infected on the pectoral fins and lateral body skin of parrotfish *Chlorurus sordidus*.

Parrotfishes (Labridae: Scarini) are an important component of coral reef ecosystems, and are harvested by commercial fisheries in the Okinawa region of southern Japan (Kishimoto, 1984; Shimose et al., 2019). The cysts (metacercariae) of *Scaphanocephalus adamsi* (Platyhelminthes, Trematoda, Heterophyidae) are frequently found on the pectoral fins and skin of commercially harvested parrotfishes in Okinawa (Iwata, 1997). These cysts, which are visible to the naked eye (Fig. 1), markedly reduce the commercial value of parrotfishes (Shimose et al., 2019). To improve the effective utilization of these fishery resources, data on the occurrence patterns of this parasite are useful for assessing the negative impact that these parasites have on parrotfish consumption, and to optimize the management of parrotfish stocks.

The genus *Scaphanocephalus* contains three species ––*S. expansus*, *S. australis*, and *S. adamsi*–– and a previous study on metacercarial cysts found on parrotfishes in Okinawa (i.e., *Scarus sordidus* and *S. rubroviolaceus*) identified them as *S. adamsi* (Iwata, 1997). A recent study also identified the metacercarial cysts on some parrotfishes as *S. adamsi*, and all cysts infecting parrotfishes in Okinawa are considered to belong to the same species (Katahira et al., unpublished data). Metacercarial cyst infection rate (i.e., prevalence) appears to vary among parrotfish species, but host specificity has not yet been evaluated. Coastal snails, marine fishes, and piscivorous raptors are thought to be the first intermediate, the second intermediate, and the final hosts of *Scaphanocephalus* parasites, respectively (Iwata, 1997). However, the process of this parasite infection event is not fully understood. In this study, parasite prevalence in the second intermediate host, parrotfishes, was assessed by means of a fish market survey as this permitted a large number of samples to be examined. Interspecific differences in parasite prevalence were evaluated quantitatively, and ecological factors influencing the prevalence in parrotfishes are discussed based on a review of the literature. The findings are expected to contribute to a more comprehensive understanding of species interactions in coral reef ecosystems.

## 2 Materials and methods

### 2.1 Data collection

*Scaphanocephalus* infection of parrotfishes was investigated by examining specimens at a fish auction site operated by the Yaeyama Fisheries Cooperative (24°21′N, 124°09′E) on Ishigaki Island, Okinawa, southern Japan. Parrotfishes are caught mainly by spearfishing at night, and small numbers are also caught by the gillnet fishery around Yaeyama Islands (Shimose et al., 2019). The market survey was conducted two or three days a week between November 2011 and October 2017 (six years), and comprised 740 days in total. The number of fish recorded did not fluctuate markedly among years or seasons. Parrotfishes being auctioned were identified to species, body weight was estimated, and parasite prevalence was assessed. The auction unit comprised a box containing one or more fishes. Basically, similarly sized individuals are placed into the same box for auction. Therefore, individual fish body size (assessed as in weight in kilograms) was estimated based on the weight of the auction box divided by the number of fish in the box. Parasite infection on fins and skin was checked by staff of the fisheries cooperative, and infected fish were placed into a same box before the start of the auction. Infected parrotfishes were occasionally found among uninfected parrotfishes, but the first author correctly recorded prevalence in these cases.

### 2.2 Estimation of parasite prevalence

The parasite prevalence (number of fish infected/number of fish recorded; Bush et al., 1997) of individuals can be explained by a logistic model with the component of each parrotfish species being the only explanatory variable. The components to which each parrotfish species belonged was modeled using a finite mixture distribution model (Leisch, 2004). This model was used to classify the parasite prevalence data for each parrotfish species. The Expectation-Maximization algorithm was then used to identify the components. This finite mixture regression model, which was implemented to assess parasite prevalence, was a mixture of binomial distributions with *s* components, where the likelihood of the *i*th observation is given by:

(1) $$H\left(p_{i}\left|\alpha \right)=\sum_{j=1}^{s}\pi_{j}Bi\left(p_{i}\right|\theta_{j}\right)$$

Here, *H* denotes the mixture probability, *j* denotes the component number, πdenotes the mixing proportion ($\sum_{j=1}^{s}\pi_{j}=1$), *Bi* denotes the binomial distribution, α denotes all parameters, $p_{i}$ denotes the parasite prevalence, and $\theta_{j}$ denotes the estimated probability of parasite prevalence for the *j*th component. The likelihood was given by the product of Eq. (1) for all observations.

The Bayesian information criterion (BIC) for the finite mixture models was compared with 2–7 components. For the best fit of the component models, the estimated probability of parasite prevalence for each component was investigated and the components were ranked by the estimated probability. Analyses were performed using R ver. 1.0.136 (R Development Core Team, 2008) and the flexmix library ver. 2.3.13 (Leisch, 2004) was used for finite mixture regression analysis.

### 2.3 Detection of ecological factors affecting parasite prevalence

To evaluate the ecological factors that could potentially affect parasite prevalence, the following information was collected for each parrotfish species from the literature, current research by the authors, and interviews with fishermen: 1) extent of scarring on corals or coral reefs as a result of feeding (scarring observed, scarring occasionally observed, and scarring not observed; Bellwood and Choat, 1990); 2) diet (epilithic algae, algae with live coral, or algae with sand surface; Bellwood and Choat, 1990); 3) potential angling target as an adult (Joh, 2001; Konishi and Nakabo, 2007; WEB-sakanazukan, 2020; personal observations); 4) use of mucous cocoons at night (interviews with fishermen).

## 3 Results

### 3.1 Parasite prevalence in parrotfishes

A total of 59,475 individual parrotfishes comprising 30 species in six genera were recorded (Table 1). The number of fishes recorded did not fluctuate markedly among years or seasons. However, the number of individuals of particular species ranged from 11,553 *Chlorurus microrhinos* individuals to only one *Ch. capistratoides* individual. Body weights also differed among species, ranging from a mean (±SD) of 0.33 (±0.03) kg in *Scarus spinus* to 1.88 (±3.51) kg in *Bolbometopon muricatum*. *Scaphanocephalus adamsi* infection was confirmed in all parrotfish species except for *Ch. bleekeri*, but this was considered to be due to the small sample size (n = 3). Two parrotfish species with a parasite prevalence of 0% (n = 3, *Ch. bleekeri*) and 100% (n = 1, *Ch. capistratoides*) were excluded from subsequent analysis.

**Table 1:** Number of individuals recorded and infected by parasites in parrotfishes (Scarini spp.) with body size information at Yaeyama fish auction site between November 2011 and October 2017. Parasite prevalences are shown for species with sample size >5. Body size is estimated as box weight divided by the number of fish included in the box.

Species (alphabetical order)	Number of fish recorded	Number of fish infected	Parasite prevalence	Estimated body weight (kg)			
				Mean	SD	Minimum	Maximum
*Bolbometopon muricatum*	270	1	0.4%	1.88	3.15	0.38	30.00
*Calotomus carolinus*	33	8	24.2%	0.39	0.10	0.24	0.70
*Cetoscarus bicolor*	1856	38	2.0%	1.21	0.59	0.26	3.50
*Chlorurus bleekeri*	3	0		0.85	0.44	0.34	1.10
*Chlorurus bowersi*	416	18	4.3%	0.44	0.11	0.25	1.20
*Chlorurus capistratoides*	1	1		0.48		0.48	0.48
*Chlorurus frontalis*	272	6	2.2%	1.01	0.44	0.23	2.90
*Chlorurus japanensis*	19	1	5.3%	0.51	0.12	0.28	0.73
*Chlorurus microrhinos*	11,553	259	2.2%	1.14	0.71	0.23	5.80
*Chlorurus oedema*	15	3	20.0%	1.20	0.62	0.35	2.50
*Chlorurus sordidus*	1026	135	13.2%	0.40	0.09	0.22	0.95
*Hipposcarus longiceps*	8512	11	0.1%	0.94	0.50	0.24	4.30
*Scarus chameleon*	13	5	38.5%	0.34	0.04	0.23	0.40
*Scarus dimidiatus*	1491	32	2.1%	0.42	0.09	0.23	0.90
*Scarus festivus*	811	116	14.3%	0.61	0.19	0.23	1.20
*Scarus forsteni*	7373	198	2.7%	0.54	0.14	0.18	1.70
*Scarus frenatus*	981	16	1.6%	0.74	0.26	0.21	1.55
*Scarus ghobban*	6441	1716	26.6%	0.67	0.33	0.20	3.50
*Scarus globiceps*	9	1	11.1%	0.36	0.11	0.27	0.57
*Scarus hypselopterus*	322	27	8.4%	0.39	0.08	0.25	0.80
*Scarus niger*	824	12	1.5%	0.45	0.10	0.23	0.97
*Scarus oviceps*	1368	12	0.9%	0.49	0.13	0.22	1.07
*Scarus prasiognathos*	1842	8	0.4%	0.86	0.39	0.18	3.30
*Scarus psittacus*	8	2	25.0%	0.34	0.03	0.28	0.38
*Scarus quoyi*	101	3	3.0%	0.39	0.08	0.25	0.70
*Scarus rivulatus*	10,450	159	1.5%	0.53	0.13	0.18	1.40
*Scarus rubroviolaceus*	2797	935	33.4%	1.38	0.57	0.29	4.10
*Scarus schlegeli*	645	145	22.5%	0.42	0.10	0.23	0.90
*Scarus spinus*	17	4	23.5%	0.33	0.03	0.28	0.39
*Scarus xanthopleura*	6	1	16.7%	1.05	0.28	0.70	1.50
Total	59,475	3873	6.5%	0.80	0.56	0.18	30.00

Parasite prevalence differed markedly among the remaining 28 host parrotfish species (Table 2). A comparison among six groupings (2–7 groups), operation into 4 groups reduced BIC the most. Host parrotfishes were ranked into the following four groups based on parasite prevalence; high: highly infected (20.0–38.5%, 8 spp.), moderate: moderately infected (8.4–16.7%, 4 spp.), low: rarely infected (0.9–11.1%, 13 spp.), and no: almost never infected (0.1–0.4%, 3 spp.) (Table 2). The lower ranking of *S. globiceps* despite this species having a higher parasite prevalence (11.1%) than *S. hypselopterus* (moderately infected, 8.4%) was due to the limited sample size for the former species (n = 9).

**Table 2:** Rank group by parasite prevalence, taxonomic (based on genus) and ecological information of 28 major (n > 5) parrotfishes (Scarini spp.) recorded at Yaeyama fish auction site. Feeding mode is from Bellwood (1994). Scarring and diet are from Bellwood and Choat (1990); E: epilithic algae, E (LC): E with live corals, E(S): E with sand surface. Angling target is from Joh (2001), Konishi and Nakabo (2007), WEB-sakanazukan (2020), and personal observation. Mucous cocoon making behavior is based on the interviews with fishers in Yaeyama. *Prevalence and rank are reversed because of sample size.

Species (infection rate order)	Parasite prevalence	Genus group	Feeding mode	Scarring (excavating)	Diet	Angling target	Mucous cocoon
Rank I (High)							
*Scarus chameleon*	38.5%	S	Scraping	No	E(S)	Yes	No
*Scarus rubroviolaceus*	33.4%	S	Scraping	Occ.	E	Yes	No
*Scarus ghobban*	26.6%	S	Scraping	Occ.	E	Yes	No
*Scarus psittacus*	25.0%	S	Scraping	No	E(S)	Yes	Yes
*Calotomus carolinus*	24.2%	Ca	Browsing			Yes	No
*Scarus spinus*	23.5%	S	Scraping	No	E	No	Yes
*Scarus schlegeli*	22.5%	S	Scraping	No	E(S)	Yes	Yes
*Chlorurus oedema*	20.0%	Ch	Excavating			Yes	Yes
Rank II (Moderate)							
*Scarus xanthopleura*	16.7%	S	Scraping			Yes	No
*Scarus festivus*	14.3%	S	Scraping			Yes	Yes
*Chlorurus sordidus*	13.2%	Ch	Excavating	Yes	E	Yes	Yes
*Scarus hypselopterus*	8.4%*	S	Scraping			Yes	Yes
Rank III (Low)							
*Scarus globiceps*	11.1%*	S	Scraping	No	E	Yes	No
*Chlorurus japanensis*	5.3%	Ch	Excavating	Yes	E	No	Yes
*Chlorurus bowersi*	4.3%	Ch	Excavating			Yes	Yes
*Scarus quoyi*	3.0%	S	Scraping			Yes	No
*Scarus forsteni*	2.7%	S	Scraping	No	E	Yes	No
*Chlorurus microrhinos*	2.2%	Ch	Excavating	Yes	E (LC)	Yes	Yes
*Chlorurus frontalis*	2.2%	Ch	Excavating	Yes	E	No	Yes
*Scarus dimidiatus*	2.1%	S	Scraping	No	E	Yes	No
*Cetoscarus bicolor*	2.0%	Ce	Excavating	Yes	E (LC)	No	No
*Scarus frenatus*	1.6%	S	Scraping	No	E	Yes	No
*Scarus rivulatus*	1.5%	S	Scraping	No	E (LC)	Yes	No
*Scarus niger*	1.5%	S	Scraping	No	E	Yes	No
*Scarus oviceps*	0.9%	S	Scraping	No	E	No	No
Rank IV (No)							
*Scarus prasiognathos*	0.4%	S	Scraping			No	No
*Bolbometopon muricatum*	0.4%	B	Excavating	Yes	E (LC)	No	No
*Hipposcarus longiceps*	0.1%	H	Scraping	No	E	No	No

### 3.2 Ecological factors influencing parasite prevalence

In the assessment of the effect of feeding behavior on parasite prevalence, “high” prevalence was observed in species in which scarring was “occasional and “no”. In the assessment of the effect of diet on parasite prevalence, parasite prevalence was “high” in parrotfish species that fed on “epilithic algae with sand surface” and “low” or “no” in species that fed on “epilithic algae with live coral”. Target angling species showed higher parasite prevalence than no target species. Mucous cocoon production did not appear to affect parasite prevalence.

## 4 Discussion

Although the prevalence of the metacercarial cysts differed markedly among host species, almost all species were infected by the parasite. A previous qualitative investigation of 119 species in 49 families of reef fishes from Okinawa showed that only eight species in four families (Aulostomidae, Mullidae, Labridae, and Tetraodontidae) were infected by *S. adamsi* (Iwata, 1997). Their results imply that the parasite exhibits weak host specificity; i.e., the parasite can infect some host fish species but prefers specific host species. The present study also showed the existence of host specificity in this parasite, even among hosts within the same tribe Scarini. This specificity may be related to host taxonomy (i.e., physical/physiological tolerance to the parasite; Loerch et al., 2015), ecological traits of host species (e.g., habitat preference, feeding ecology; Sikkel et al., 2009; Elmer et al., 2019), or factors related to taxonomy and feeding ecology (Sikkel et al., 2018).

**Fig. 2:**
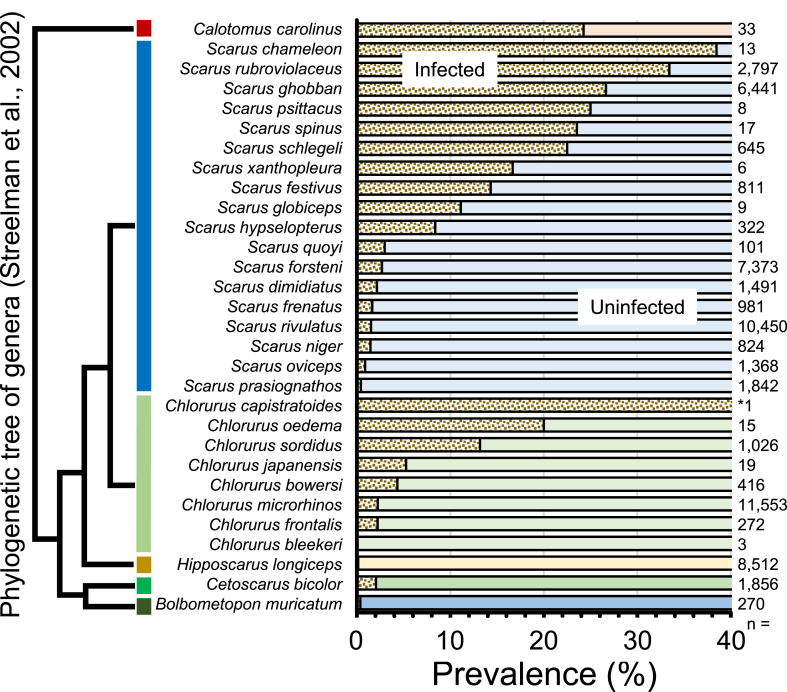
Phylogenetic tree of genera in Scarini of Labridae (modified from Streelman et al., 2002) and parasite prevalence in each species. *: 100%.

The parrotfishes examined in the present study belonged to six genera (Bellwood, 2001). Of the six genera, *Calotomus* (1 sp. in this study) was the outermost group (Streelman et al., 2002) and was highly infected (Fig. 2). Conversely, the genera *Bolbometopon* (1 sp.), *Cetoscarus* (1 sp.) and *Hipposcarus* (1 sp.) were rarely infected. The genera *Scarus* and *Chlorurus* are considered to be sister groups (Streelman et al., 2002) and are highly divergent among parrotfishes (Bellwood, 1994); parasite prevalence in these genera differed markedly among species (Table 2). These results suggest that parasite prevalence in parrotfishes was not affected by phylogenetic relatedness (Streelman et al., 2002) or feeding mode among host species (feeding mode linked to genus; Bellwood, 1994). In addition, parasite prevalence was also found to be independent of host size.

A variety of ecological factors can affect parasite prevalence in parrotfishes. These factors act as encounter filters that determine whether the infective cercariae that are released from the first intermediate hosts successfully reach the second intermediate fish hosts (Combes, 1991; Poulin, 2007). Possible filters include the diurnal and nocturnal habitats used by the host parrotfishes, and whether or not they construct a mucous cocoon when they sleep at night. The habitat preferences of parrotfishes have been studied extensively, with most species inhabiting the shallow areas, around reefs, lagoons and drop-offs (Bellwood, 2001; Katoh, 2016); however, no clear relationships between habitat utilization and parasite prevalence in parrotfish was detected.

Constructing mucous cocoons to sleep at night is a well-known parrotfish behavior (e.g., Casimir, 1971; Kishimoto, 1984). The cocoons have been shown to protect the parrotfish against attacks by gnathiid isopods, which feed on fish blood (Grutter et al., 2011). Other possible functions of the cocoon include avoiding predation by moray eels (Winn and Bardach, 1959) and/or to protect the parrotfishes from infection by *Scaphanocephalus* parasites. According to fishermen on Ishigaki Island who target parrotfishes at night, *Calotomus*, *Hipposcarus*, and some *Scarus* species do not make cocoons, while all *Chlorurus* species and some *Scarus* species do (Table 2). In this study, the parrotfishes that made cocoons, such as *S. festivus* (14.3%) and *C. sordidus* (13.2%), were found to be moderately infected, whereas parrotfishes that did not make cocoons, such as *S. prasiognathos* (0.4%) and *H. longiceps* (0.1%), were almost never infected. These results suggest that the cocoons do not play an important role in protecting parrotfish from *Scaphanocephalus* parasites. Given that they are typically short-lived and highly susceptible to environmental perturbations (Pietrock and Marcogliese, 2003), the free-living trematode cercariae typically have a temporal pattern to be released from the first intermediate host (Combes et al., 1994). It is therefore the timing of making cocoons at night may not match the timing of release of cercariae.

As an alternative scenario for the mode of parasite infection, the range of feeding habits exhibited by parrotfish could be considered. The diet (Bellwood and Choat, 1990) and utilization of a particular target species for recreational angling (Joh, 2001; Konishi and Nakabo, 2007) are known for a variety of parrotfish with different feeding habits (Table 2). Parrotfishes in genera *Bolbometopon*, *Cetoscarus*, *Chlorurus*, *Hipposcarus* and *Scarus* generally feed on epilithic algae, and some species also feed on live corals and ingest sand surface (Bellwood and Choat, 1990). The diet of three highly infected parrotfishes, *S. chameleon* (38.5%), *S. psittacus* (25.0%), and *S. schlegeli* (22.5%), consists of epilithic algae with sand surface (Bellwood and Choat, 1990). The feeding behavior of two other highly infected parrotfishes, *S. rubroviolaceus* (33.4%) and *S. ghobban* (26.6%), can be characterized as “scarring occasionally observed” on corals (Bellwood and Choat, 1990). This scarring is referred to as being occasional because these species have a more generalist diet. On the other hand, the diet of parrotfishes that were only rarely or almost never infected, *B. muricatum* (0.4%), *S. rivulatus* (1.5%), *Ce. bicolor* (2.0%), and *C. microrhinos* (2.2%), consists of epilithic algae with live coral (Bellwood and Choat, 1990). These species also tend to occur in relatively shallow, wave-exposed locations (Bellwood et al., 2003; Fox and Bellwood, 2007) in areas with relatively clean algal turfs (Purcell and Bellwood, 2001).

*Scarus chameleon* (prevalence = 38.5%), *S. rubroviolaceus* (33.4%), *S. ghobban* (26.6%), *Calotomus carolinus* (24.2%), *S. schlegeli* (22.5%), and *S. xanthopleura* (16.7%), *S. festivus* (14.3%), *Chlorurus sordidus* (13.2%), *S. forsteni* (2.7%), *C. microrhinos* (2.2%), and *S. rivulatus* (1.5%) are listed in two field identification guides as being targets of recreational anglers in the Okinawa region (Joh, 2001; Konishi and Nakabo, 2007). The bait used to catch these species includes euphausiid krill and hermit crabs (Joh, 2001), implying that these species have partially common feeding habits. In addition, with the exception of three species (*S. forsteni*, *C. microrhinos*, and *S. rivulatus*), many of these parrotfishes were found to be highly or moderately infected. Of the three exceptions, two species include live coral in their diet (Table 2). In addition, almost all of the parrotfishes that are almost never infected, i.e. *S. prasiognathos* (0.4%), *B. muricatum* (0.4%), and *H. longiceps* (0.1%), are not targets of recreational anglers despite being highly abundant (Table 1). Similarly, parrotfishes that were only rarely infected also tended not to be the targets of recreational angling.

These findings imply that feeding ecology appears to be most strongly associated with parasite prevalence in parrotfishes. For example, the parrotfish species that tend to cause occasional scarring in corals, ingest the sand surface, and are caught by angling using crustacean baits, tend to be infected more. Those species that feed on live corals and are not caught by anglers tend to be less infected. However, metacercarial infection is thought to occur via external routes, such as through penetration of the fins or skin of the host, and not through ingestion of prey items (Cribb, 2005). Therefore, the relationship between feeding ecology and parasite prevalence appears to be indirect. It also appears that parrotfish feeding behavior in habitats where the first intermediate host (e.g., snails) occurs (Iwata, 1997), or in which the pelagic stage of *Scaphanocephalus* occurs, are the primary factors affecting parasite prevalence in parrotfishes. At this stage of development, the parasite may occur at high densities and have a localized distribution compared to other areas. For example, if the parasite is concentrated near the sand surface or in the habitats of the krill-like crustaceans that are preyed upon by the targeted parrotfishes, then the interspecific differences in parasite prevalence among parrotfish species could be explained. Similarly, it is possible that the parasite is rare near the live corals that are preyed upon by some parrotfish species. The prevalence of the congeneric *Scaphanocephalus expansus* is known to vary as a function of habitat characteristics (i.e., depth and area), even in the same host species (e.g., *Acanthurus tractus*, Elmer et al., 2019). In future studies, parasite prevalence in parrotfishes should therefore also be examined among different habitats.

In conclusion, the prevalence of *Scaphanocephalus* in parrotfishes is considered to be linked to the feeding ecology of the host, and not to taxonomic affiliation or cocoon-making behavior. The life history and infection process for *Scaphanocephalus* parasites are still poorly understood. Ecological traits of the host fish species and detailed studies of their feeding behavior will contribute to a more comprehensive understanding of parasite-host relationships in coral reef ecosystems.

## Declaration of competing interest

On behalf of all authors, the corresponding author states that there is no conflict of interest.
